# Teaching/learning formats and cross-cutting issues for the design of interprofessional education for healthcare professions – literature review and analysis of training and examination regulations

**DOI:** 10.3205/zma001750

**Published:** 2025-04-15

**Authors:** Jann Niklas Vogel, Annemarie Bagner, Rebecca Schnaak, Matthias Müller

**Affiliations:** 1 Neubrandenburg University of Applied Sciences, Department of Social Work, Education and Childcare, Neubrandenburg, Germany

**Keywords:** interprofessional learning, interprofessional education, interprofessionalism, healthcare training, interprofessional collaboration, skills

## Abstract

**Background:**

Educational institutions in Germany are facing the challenge of providing healthcare professionals with the skills they need to collaborate interprofessionally. Appropriate skills can be acquired through suitable teaching/learning formats and cross-cutting topics. However, there is a lack of empirical results on the design of appropriate teaching/learning formats in order to apply the cross-cutting topics effectively in as many healthcare professions as possible.

**Methodology:**

An integrative literature review on suitable teaching/learning formats was carried out in which 21 titles were analysed and presented in a table. The typology according to Sottas et al. was used as a deductive evaluation framework. In order to identify cross-cutting issues, an analysis of the training and examination regulations for healthcare professions in Germany was carried out, with an evaluation using frequency counts.

**Results:**

The three most common cross-cutting topics are counselling, quality assurance and the structured care process. The topics are to be taught using methods such as case-based and problem-oriented learning, interprofessional group work or simulation. The debriefing of interprofessional teaching/learning formats is highly relevant.

**Discussion & conclusion:**

Interprofessional teaching for healthcare professions rarely takes place in the form of observation-based and hands-on learning in Germany. In addition, only a few healthcare professions are usually involved in interprofessional teaching and learning formats. Both of these factors impede the teaching of complex interprofessional skills.

## Zusammenfassung

**Hintergrund:**

Bildungseinrichtungen in Deutschland stehen vor der Herausforderung, Gesundheitsfachberufe zur interprofessionellen Zusammenarbeit zu qualifizieren. Entsprechende Kompetenzen können über geeignete Lehr-/Lernformate und Querschnittsthemen angebahnt werden. Allerdings fehlen empirische Ergebnisse zur Gestaltung entsprechender Lehr-/Lernformate, um die Querschnittsthemen effektiv in möglichst vielen Gesundheitsfachberufen anzuwenden.

**Methodik:**

Es wurde eine integrative Literaturübersicht zu geeigneten Lehr-/Lernformaten durchgeführt, in der 21 Titel inhaltsanalytisch ausgewertet und tabellarisch dargestellt wurden. Dabei diente die Typologie nach Sottas et al. als deduktiver Auswertungsrahmen. Zur Identifizierung von Querschnittsthemen wurde eine Analyse der Ausbildungs- und Prüfungsordnungen der Gesundheitsfachberufe in Deutschland durchgeführt, die über Häufigkeitszählungen ausgewertet wurden.

**Ergebnisse:**

Die drei häufigsten Querschnittsthemen bilden Beratung, Qualitätssicherung und der strukturierte Versorgungsprozess. Die Themen sollten über Methoden wie fallbasiertes und problemorientiertes Lernen, interprofessionelle Gruppenarbeit oder Simulation vermittelt werden. Einer hohen Relevanz kommt der Nachbesprechung interprofessioneller Lehr-/Lernformate zu.

**Diskussion & Schlussfolgerung:**

Interprofessionelle Lehre für Gesundheitsberufe erfolgt in Deutschland kaum in Form des beobachtungsbasierten und handlungsbasierten Lernens. Zudem sind meist nur wenige Gesundheitsberufe an interprofessionellen Lehr- und Lernformaten beteiligt. Beides steht einer Vermittlung komplexer interprofessioneller Kompetenzen entgegen.

## 1. Background

The German healthcare sector faces complex challenges in light of ongoing developments such as demographic change, an increase in chronic illnesses and co- and multimorbidity, technical innovations and the academic approach of healthcare professions [1], [2], [3]. These challenges are so complex that they cannot be dealt with on a single-profession basis. International literature therefore describes interprofessional healthcare teams as the key to professional, needs-based, people-centred and cost-effective healthcare [4], [5], [6], [7], [8]. The World Health Organization (WHO) defines interprofessionalism as teaching and work that occurs when at least two professional groups work together and learn from each other [9]. In addition to direct patient care, such interdisciplinary thinking and action also affects the overarching planning and coordination of care processes [3]. This requires skills which include, inter alia, knowledge of the strengths and abilities of other healthcare professions. According to Sottas et al., the taxonomically higher-ranking interprofessional core competences consist of interprofessional communication, functioning as a team, clarification of roles and responsibilities, joint decision-making, conflict resolution and continuous quality improvement [2]. Appropriate skills can be developed in the initial, continuing and further training of healthcare professionals through interprofessional teaching and learning (IPTL) [3], [10]. IPTL seems particularly relevant for professional training in order to introduce young professionals to the cooperative practice of healthcare work as early as possible [11]. However, educational institutions are faced with the challenge of creating an appropriate framework for IPTL. There are now projects for the practical implementation of IPTL, but these have rarely been scientifically monitored and/or adequately evaluated [12]. There are therefore barely any systematic findings on IPTL in German-speaking countries. Internationally, the systematic review by Aldriwesh et al. provides an overview of IPTL teaching/learning formats [13]. According to Aldriwesh et al., the key for IPTL is planning by members of various professional groups, IPTL implementation in the form of an interactive process and collaboration as an essential learning outcome, in which reflection plays a key role [13]. The review identifies simulation-based learning, e-learning and problem-based learning as the most frequently mentioned teaching/learning formats for IPTL [13]. 

Generally speaking, German-speaking countries cannot keep up with internal experience with IPTL [11]. For example, in English-speaking and Scandinavian countries, there are numerous theories, models and structures that provide a country-specific framework for establishing interprofessional education for healthcare professionals. In Canada, the National Interprofessional Competency Framework sets out four central areas of healthcare competence [14]. Key elements include collaborative patient-centred care and interprofessional communication with other healthcare professionals [14]. The Competency Framework can be used by individuals or organisations to integrate interprofessional collaboration into education and professional practice. In the context of vocational training, the Competency Framework constitutes a starting point for structuring and planning healthcare education [14]. In addition to Canada, the USA also has a catalogue of competencies for healthcare education and practice published by the Interprofessional Education Collaborative (IPEC). The IPEC catalogue of competencies is also made up of four domains of competencies, including 33 statements on sub-competencies [6]. To summarise, the international competency frameworks make it clear that there are cross-cutting topics for healthcare professions that enable learners to learn interprofessionalism in direct theory-practice transfer [15]. 

In Germany, IPTL has to be developed and established for healthcare professions [16]. The teaching of IPTL is based on cross-cutting topics, i.e. topics which are relevant to several professional groups in professional activities [17]. The cross-cutting topics should teach specialist knowledge and competencies enabling learners to learn from one another, about one another and with one another [17]. But how are teaching/learning formats for interprofessional healthcare education in Germany designed and what cross-cutting topics affect as many healthcare professions as possible? A sub-project of the joint project Campus BWP MV^1^, the University of Rostock and the University of Applied Sciences Neubrandenburg addressed these questions. Design aspects of interprofessional teaching and recommendations for action are derived based on the research results.

## 2. Method

An integrative literature search was carried out. This methodology provides the opportunity to consider theoretical and empirical, qualitative and quantitative research methods so that the current state of research on the issue can be demonstrated and theories can be developed and areas of application identified on this basis [18]. The SPIDER scheme was used to operationalise the research question [19] (see table 1 (Tab. 1)).

The starting point was the search in MEDLINE via PubMed as the central database for scientific studies in healthcare professions. The PubMed search supported the generation of suitable mesh terms for the research question and was adapted to the ERIC databases and Web of Science Core Collection [20] (see table 2 (Tab. 2)). ERIC covers the educational discourse on the research question and Web of Science Core Collection combines the educational and health professional focal points of the research question in one database. 

**Figure 1:**
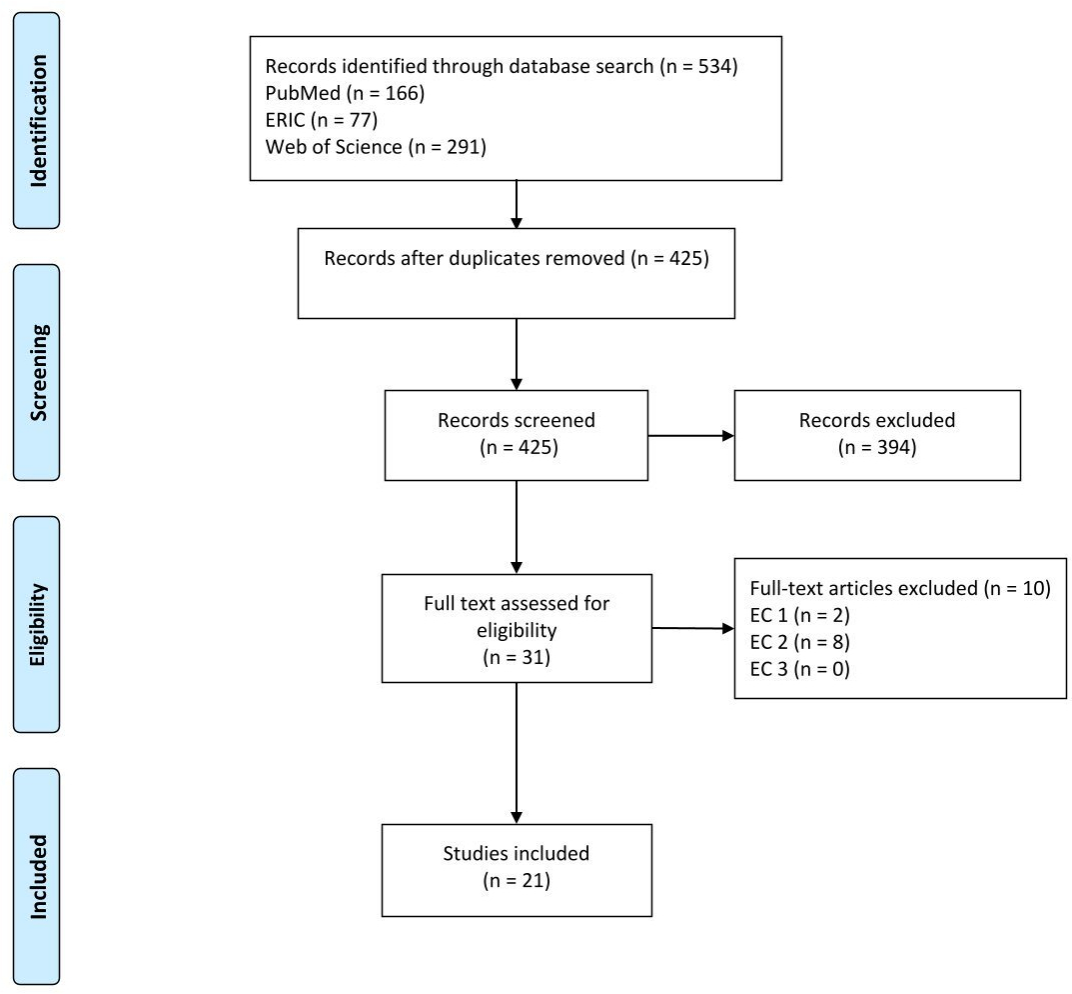
Flow diagram of the literature search (based on [https://www.prisma-statement.org/prisma-2020-flow-diagram]) Note: n: number, EC: exclusion criteria, EC 1: lack of reference to at least two healthcare professions, EC 2: no reference to the research question, EC 3: the publication is not based in Germany or no reference is established to the German healthcare system

The literature search took place in the period from 01/01/2023 to 30/04/2023. The documentation of the search is based on the PRISMA statement [21] (see figure 1 (Fig. 1)). Two researchers used the search strings to search the databases independently and reviewed all studies for relevance and suitability. The following studies were considered: German and English studies, which address the teaching/learning formats of IPTL in training and/or study for healthcare professions in Germany. In this regard, a geographic filter was not used in the search string in order to guarantee as comprehensive a selection as possible of potentially relevant studies. The geographic selection was made using database-specific filters and as part of the title and abstract screening. After the title abstract screening, the researchers compared their results. Any contradictions or discrepancies in the evaluation were clarified through discussion and consensus. Studies matching the inclusion criteria were used for the full text evaluation. This was also carried out by two researchers. In seven studies, the authors were contacted in order to obtain further information. In two studies, no further data could be obtained. A consensus meeting took place to select the studies, in which the four authors agreed on the inclusion of the studies. The data was then extracted for each study included. Qualitative content analysis was used to form categories in an inductive-deductive alternation [22]. Categories are derived directly from the material in the inductive formation of categories [23]. The deductive formation of categories defines the categories before the data analysis [23] and refers to the SPIDER scheme used. The typology according to Sottas et al., which classifies teaching/learning formats of IPTL into six successive stages was used as the deductive evaluation framework [11]. The quality of the teaching/learning formats increases with higher categories [11] (see table 3 (Tab. 3)). The typology illustrates that IPTL must first teach fundamentals such as perception, appreciation, communication and understanding in order to create the prerequisites for the acquisition of taxonomically higher interprofessional skills [11]. Practice-based learning is required for taxonomically higher skills [11].

Overall, five categories were formed for the data analysis (see attachment 1 ). These were “topic”, “teacher”, “student”, “time frame” and “classification based on Sottas et al. [11]”. The latter specifies the IPTL design used.

The literature search was expanded to identify cross-cutting topics in healthcare professions in Germany. To this end, the training and examination regulations for healthcare professions in Germany were analysed. This procedure was selected because the public availability and nationwide regulations provide greater thematic coverage compared to the usually state-specific framework curricula for vocational schools. The analysis was carried out by two researchers from April 2023 to May 2023. The website “Gesetze im Internet” [https://www.gesetze-im-internet.de/] was used to search for the training and examination regulations of the following healthcare professions:

1. Nurse
2. Physiotherapist
3. Dietician
4. Occupational therapist
5. Midwife/obstetrician
6. Speech therapist
7. Masseur and masseuse as well as hydrotherapist
8. Medical technician
9. Emergency paramedic
10. Orthopaedic practitioner
11. Podiatrist
12. Pharmaceutical assistant

The data was transferred to Microsoft Excel and organised in a table in accordance with the professional nursing regulations. Due to the amendments to the Nursing Professions Act, the professional regulations offered a particular level of differentiation and relevance to the current substantive discussion of IPTL. The categories were formed deductively with reference to the Training and Examination Regulations for Nursing Professions (PflAPrV) (see table 4 (Tab. 4)). The categories formed were then counted [24], [25].

## 3. Results

The literature search resulted in 10,290 identifications, of which 425 results were included in the pre-selection. After screening the titles and abstracts, 31 titles were included in the full text search. Overall, 21 titles met the inclusion criteria and were included in the integrative literature review (see figure 1 (Fig. 1)). 

10 of these texts are in English and 11 texts are in German or available in a German version. The oldest title included was published in 2006. Each of the titles included addresses teaching/learning formats of IPTL for the training and/or study of healthcare professions in Germany. Table 5 (Tab. 5) provides an overview of the titles included. The contents of the titles are present in attachment 1 . In general, the titles have considerable differences in terms of the methodology and duration of the IPTL interventions. The period of intervention ranged from a total of 120 minutes [26], [27], [28] to a course of two semester hours per week [29]. IPTL most frequently takes place between vocational students in medicine (n=18) and nursing (n=15). IPTL takes place with more than two healthcare professions in seven of the 21 titles included. IPTL is usually designed and implemented on a practical level by members of different healthcare professions. It is clear that although IPTL can be taught by one professional group in the titles included, it is aimed at at least two professional groups of students. Lessons are often organised using the methodology of case-based teaching and learning, with simulations being used [30], [31], [32], [33], [34], [35]. 

The training and examination regulations (N=12) were analysed with reference to the German Qualification Framework for Lifelong Learning (DQR) [36] and the thematic areas of the PflAPrV. The frequency of common training content was determined on the basis of the competence dimensions (see table 4 (Tab. 4)). 

The areas of “counselling”, “quality assurance”, “structured care process”, “team work” and “hygiene” were identified as relevant cross-cutting issues, with the area of “counselling” being the most frequently represented in the training and examination regulations in percentage terms. The areas of social skills or developing a professional identity are represented less in percentage terms.

## 4. Discussion

The results can be differentiated into the framework conditions for and the content of IPTL. In terms of the framework conditions for IPTL, it can be noted that the didactic and content-related conception as well as the application of IPTL often takes place in collaboration with several healthcare professions. Even if this is a constructive element of IPTL, it must be emphasised because there are certainly alternative movements in practice and attempts are being made to develop and apply IPTL on a mono-professional basis. IPTL is often implemented between vocational students in medicine (n=19) and nursing (n=15) (see also [12]). The following three cross-cutting issues are in particular relevant: “counselling”, “quality assurance” and “structured care process”. The cross-cutting issues mentioned are most likely to ensure that there is interprofessional interest in IPTL. This interpretation is also confirmed by the international competency frameworks of the USA and Canada [6], [7], which place a similar emphasis on communication, collaboration and role clarification, which is key to structured care and quality assurance. It should be emphasised that according to Sottas et al., the basics (appreciation, perception, understanding and communication) must first be taught in order to develop taxonomically higher-value interprofessional core competences [2]. Such a fundamental inner attitude, embodied by professional identity and social skills, is not emphasised much in the training and examination regulations of the healthcare professions in Germany, which can be attributed to various causes. On the one hand, the training and examination regulations focus heavily on occupation-specific knowledge and technical expertise as these form the basis of patient safety and care quality. On the other hand, the training and examination regulations are geared towards teaching specific measurable skills whereas social skills and professional identity are difficult to operationalise and evaluate. For these reasons, the three identified cross-cutting issues in this work are found to be suitable for designing IPTL. The cross-cutting issues provide a basis for different professions to contribute their perspectives and learn from one another, whereby integrated problem solving and team work are promoted. The present study results show that IPTL applications range in duration from a total of 120 minutes [27], [28], [29] to a course of two semester hours per week [30]. The implementation period is heavily dependent on the application model (see attachment 1 ) and therefore presumably results more from the content intention of the IPTL. The implementation variant of IPTL (e.g. block course, excursion, simulation training) seems particularly important here. The case study, for example by creating case scenarios, seems to be the method of choice with IPTL, although simulations are also used [31], [32], [33], [34], [35], [36]. These results are consistent with the systematic review work of Aldriwesh et al., in which simulation-based learning, e-learning and problem-based learning were identified as the most frequently cited teaching/learning formats for IPTL [13]. 

The content design of IPTL should be geared towards the typology in accordance with Sottas et al. Several classification levels can be used if different teaching formats are used. These results indicate that IPTL takes place most often (nine citations) at the low level of exchange-based learning [27], [28], [29], [30], [32], [33], [37], [38], [39]. With regard to the reasons, it is reasonable to assume that IPTL at this classification level requires the least differentiated preparation. The next relevant level which was identified with several citations is the fourth level of simulation-based learning (eight studies [31], [33], [34], [35], [40], [41], [42], [43]. In the simulation, there is the opportunity to teach more differentiated skills and more demanding requirement levels via IPTL. The sixth and highest classification level of IPTL realistically depicts the later working environment (four studies [36], [44], [45], [46]) IPTL therefore seems even suitable for training complex professional situations. 

Overall, the results can be differentiated in a triad of basic qualification, thematic specialisation and complex combination of professional practice. It is therefore also logical that there were no findings on the suitable timing of IPTL in training and studies [11], [47]. It remains unclear how to interpret the gaps in implementation of the third and fifth level. It is possible that IPTL in the third and fifth classification levels is associated with a high degree of organisational effort, which is why simulation-based learning and practice-based learning could be used more in vocational training. In terms of group size, three of the studies included [30], [40], [43] explicitly report small group work as part of IPTL. This is consistent with the requirements of Nock [17], which also recommends carrying out IPTL in small groups and avoiding large events. Even if no broad database was identified in this regard in this study, it seems obvious to implement IPTL in smaller groups, as this is more likely to ensure that the professions engage in an interprofessional exchange. Finally, the literature recommends a learning outcome-oriented backward design [48], [49] of IPTL [50], which should ensure the harmonisation of course objectives, content and tasks.

The present results have various limitations. For example, an integrative literature review like this one may result in distortions in the selection of the criteria, search methodology and data analysis. The literature search carried out here only includes German and English-language publications in selected databases. The inclusion of other languages, databases and additional search terms could lead to an increase in the literature to be included and therefore generate other key results. The methodology of the integrative review is sometimes criticised for being difficult to evaluate and interpret due to the synthesis of different research approaches [51]. However, especially against the background of the heterogeneous study landscape, the integrative potential of the review was deemed effective in order to gain the broadest possible understanding of IPTL. Nevertheless, inaccuracies may have arisen in the integrative interpretation.

## 5. Conclusion

Based on the present results, the use of IPTL depends on the appropriate time, the incorporation into teaching and the intended objective in training [11]. The incorporation of IPTL in the curriculum is of great importance, as it allows interprofessional skills to be built up over a long period of time and systematically taxonomically higher competence levels to be built upon the previous “basics” [11]. A single course or a single module are therefore not enough to develop appropriate interprofessional skills. 

The results indicate that IPTL implementation in Germany has gaps. On the one hand, IPTL often only takes place between a few healthcare professions, which precludes teaching complex interprofessional skills. On the other hand, IPTL hardly takes place in the form of observation-based learning and activity-based learning in accordance with the classification levels of Sottas et al. There is a need for increased implementation projects by educational institutions to close this gap and create the conditions for the acquisition of taxonomically higher interprofessional skills. In order to support educational institutions in this project, institution-specific implementations of IPTL as well as preferences and needs of teachers should be identified. Moreover, more detailed research into the effects of IPTL with regard to the learning outcome is still outstanding. The objective here would be to identify effective implementation variants of IPTL. The perspectives of teachers and students, data on the long-term effects of IPTL [17] and the transfer of IPTL into professional practice [51] appear particularly relevant. Cross-institutional evaluation instruments (e.g. FILE) should be used for such evaluations in order to enable the results to be compared [51]. 

Finally, legislation such as the Nursing Competence Act, the cornerstones of which were published by the Federal Ministry of Health (BMG) in 2023, could ensure greater commitment to IPTL in training and thus be beneficial for a virtuous cycle of IPTL implementation and research. 

## Note

^1^ The project Campus BWP MV aims to implement a state-wide, cross-phase overall strategy for teacher training in Mecklenburg-Vorpommern in terms of the three areas of quality assurance, quality development and co-operation and networking: individual, structure and quality. 

## Abbreviations

- IPEC = Interprofessional Education Collaborative
- IPTL = Interprofessional Teaching and Learning
- OER = Open Educational Resources
- PflAPrV = Training and Examination Regulations for Nursing Professions

## Funding

This work is part of the joint project “CAMPUS BWP MV”. The project was funded by the Federal Ministry of Education and Research as part of the joint “Teacher Training Quality Campaign” of the federal and state governments. Funding code: 01JA2023A.

## Authors’ ORCIDs

- Jann Niklas Vogel: [0000-0002-8937-6172]
- Rebecca Schnaak: [0009-0005-6127-1861]
- Matthias Müller: [0000-0001-5694-0083]

## Competing interests

The authors declare that they have no competing interests. 

## Supplementary Material

Evaluation results of the publications included

## Figures and Tables

**Table 1:**
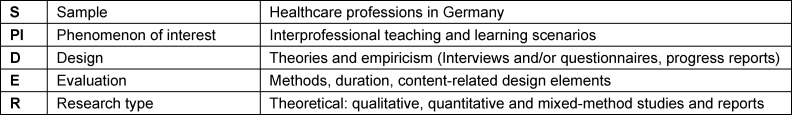
Research question using the SPIDER tool

**Table 2:**
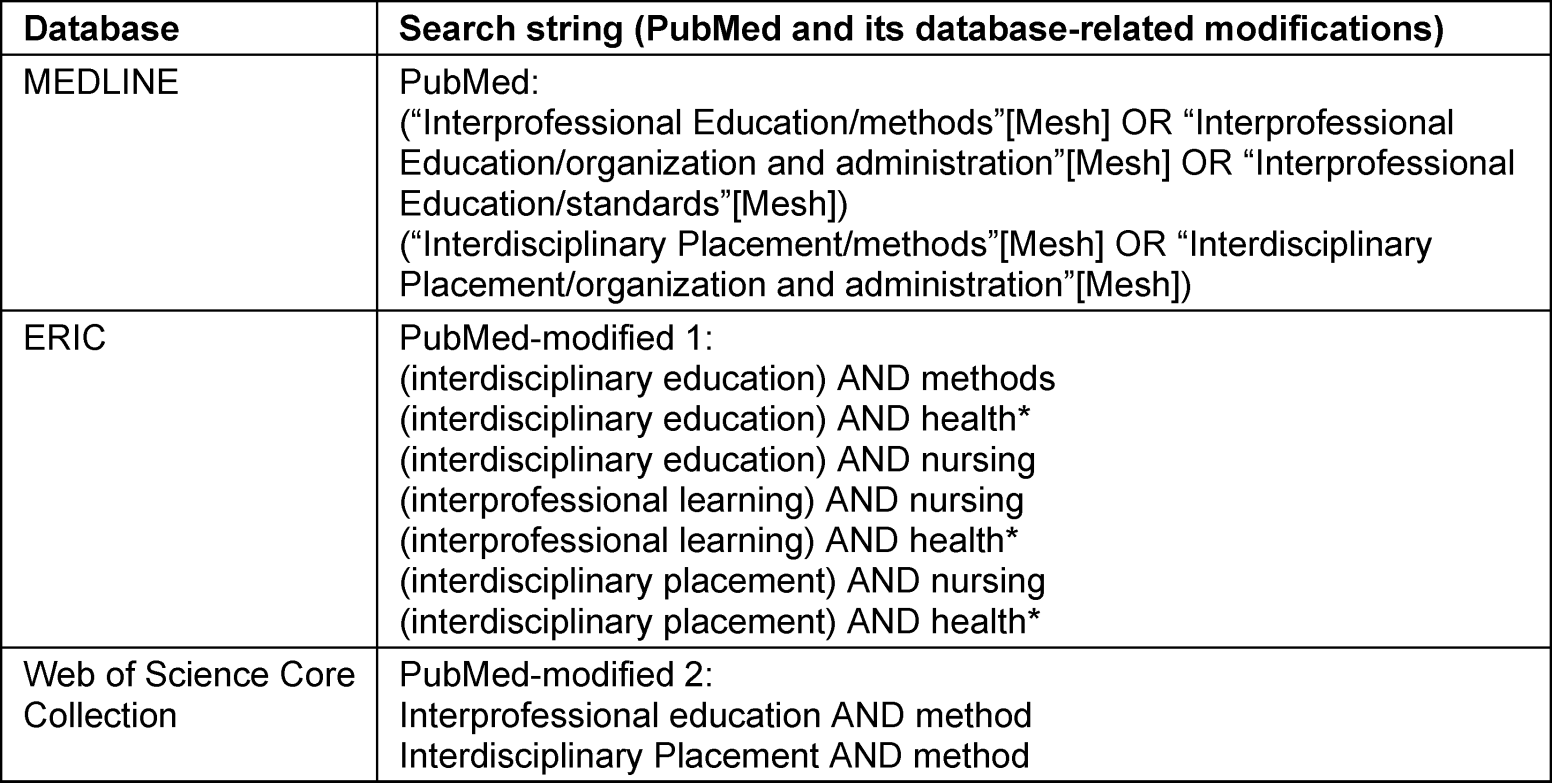
Databases and search strings used

**Table 3:**
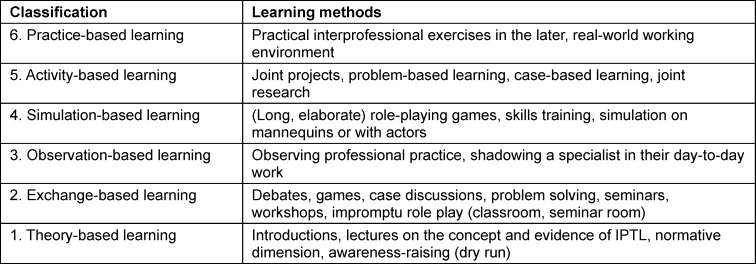
IPTL typologies (adapted from [11])

**Table 4:**
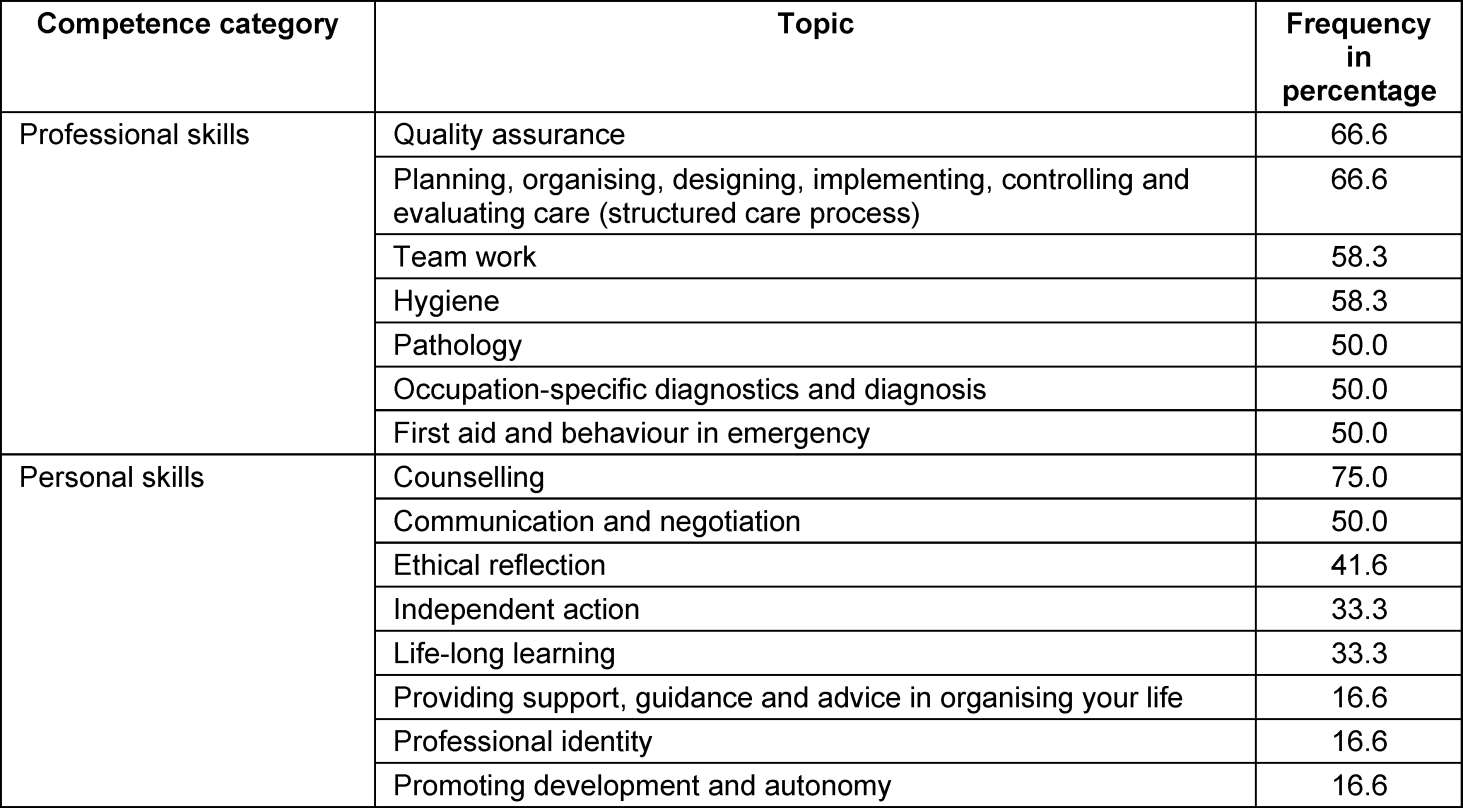
Common training content of healthcare professions in Germany

**Table 5:**
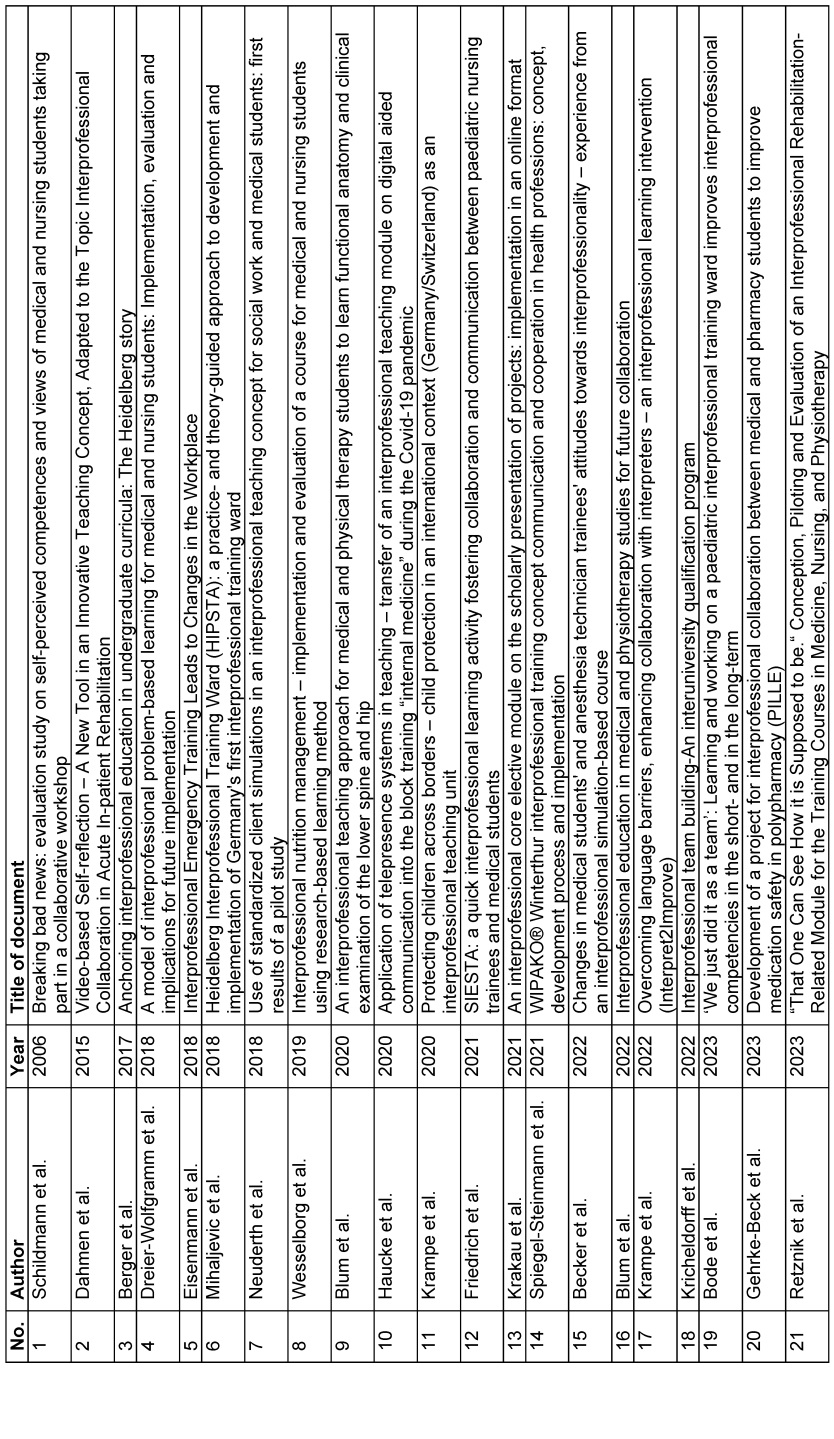
Overview of the publications included
