# Supplementary material for: Teaching/learning formats and cross-cutting issues for the design of interprofessional education for healthcare professions – literature review and analysis of training and examination regulations
Source: GMS J Med Educ. 2025 Apr 15;42(2):Doc26. doi: 10.3205/zma001750 (PMC12131511; doi:10.3205/zma001750)
Supplement: Evaluation results of the publications included [file JME-42-26-s-001.pdf]

Attachment 1: Evaluation results of the publications included

Source: own illustration

No.	Topic	Teachers	Students *(professions)*	Time frame	Classification based on Sottas et al. (2017) *(Design)*
1	Breaking bad news	Team of two healthcare professions	Medicine, nursing	8 h	2. Exchange-based learning
2	Mobilisation in early-stage rehabilitation	Rehabilitative medicine, physiotherapy, nursing, general surgery/experimental surgery	Medicine, nursing, physiotherapy	5 modules spread over 5 course days with a total of 30 TU including self-study	4. Simulation-based learning
3	4 different seminars with different topics: team communication, medical error communication, English in healthcare, small business management	ip project team (medicine, nursing, sociology)	Medicine, interprofessional health care	4 different seminars on different topics	2. Exchange-based learning
4	Community Medicine II - Job-shadowing programme	Doctor, nurse (tutorials by medical students with emergency paramedic training)	Medicine, nursing	42 TU of 45 min.	6. Practice-based learning
5	ip emergency care	The modules were planned by an ip team (medicine, nursing, paramedic)	Medicine, nursing, emergency paramedic	Several one-day training modules	4. Simulation-based learning
6	General, visceral and transplant surgery	ip organisation team, learning guides: Doctors, nursing staff	Medicine, nursing	4 weeks	6. Practice-based learning
7	Training for counselling sessions with seriously ill clients	Doctors, psychologists, social workers and lawyers	Medicine, social work	Up to 12 × 90 min.	2. Exchange-based learning; 3. Observation-based learning
8	Malnutrition	Medicine, nursing science, vocational education in nursing, ecotrophology, dietary assistance	Medicine, nursing	Course with 2 SHW, organisation over 6 block dates	2. Exchange-based learning

Attachment 1 to Vogel JN, Bagner A, Schnaak R, Müller M. *Teaching/learning formats and cross-cutting issues for the design of interprofessional education for healthcare professions – literature review and analysis of training and examination regulations*. GMS J Med Educ. 2025;42(3):Doc26. DOI: 10.3205/zma001750

No.	Topic	Teachers	Students *(professions)*	Time frame	Classification based on Sottas et al. (2017) *(Design)*
9	Functional anatomy and clinical examination of the lower spine and hip	University anatomists, orthopaedics, physiotherapy	Medicine, pysiotherapy	3 workshops of 40 min.	2. Exchange-based learning
10	ip teleconsultation	Medicine	Medicine, nursing	Block placement (3h)	4. Simulation-based learning
11	Child protection in an international context (Germany/Switzerland)	Medicine, nursing science, social work	Medicine, nursing science, social work	One day	2. Exchange-based learning
12	ip paedeatric training ward (IPAPAED)	Paediatrics, paediatric care	Medicine, nursing	4 sessions of 30 min.	2. Exchange-based learning
13	Scientific presentation of project work	Nursing, physiotherapy, speech therapy	Nursing, physiotherapy, speech therapy, orthoptics, occupational therapy, MTLA, MTRA, OTA, midwife	135 h	5. Activity-based learning
14	Communication and cooperation in healthcare professions	ip concept development	Occupational therapy, midwifery, nursing, physiotherapy, health promotion & prevention	3 modules in the 2nd, 5th and 6th semester totally 12 ECTS	2. Exchange-based learning; 4. Simulation-based learning
15	Crisis management	Anaesthetists, anaesthesia care, anaesthesia technology	Medicine, anaesthesiology	4 sessions of 3.5 h	4. Simulation-based learning
16	Anatomy in the area of the lower back and hips	Lecturers	Medicine, physiotherapy	3 sessions of 40 min.	2. Exchange-based learning
17	Dealing with language barriers, collaboration with interpreters	Paediatrician, medical psychologist specialising in communication research, head of the local interpreter pool, trainer for the simulated patients	Medicine, nursing	3 h	4. Simulation-based learning
18	Concerns and needs of geriatric patients; developing suitable treatment and intervention plans	Professors work together with subject-related teaching tandems (social work, physiotherapy, nursing)	Medicine, nursing science, vocational education in healthcare, social work	16 TU on 2 days (8 TU each) at intervals of 2 to 3 weeks	4. Simulation-based learning
19	Paediatrics	Paediatrics, paediatric care	Medicine, nursing	2 weeks	6. Practice-based learning

No.	Topic	Teachers	Students *(professions)*	Time frame	Classification based on Sottas et al. (2017) *(Design)*
20	Multimorbidity, drug safety with polypharmacy	General medicine, clinical pharmacy	Medicine, pharmacy	2 TU ip seminar,<br>2 TU ip placement in simulation pharmacy, 4 TU ip work shadowing in a GP practice	6. Practice-based learning
21	Rehabilitations and discharge management	Module conception by ip lecturer team	Medicine, nursing, physiotherapy	Two days plus online preparation	4. Simulation-based learning

Note. h=hours; ip=interprofessional; LV=course; min=minutes; MTLA=medical laboratory technologist; MTRA=radiologic technologist/radiographer; OTA=surgical technologist; SHW=semester hours per week; TU=teaching units. *Classification based on Sottas et al. (2017)*. $N_{6.\text{Practice-based learning}}$=4; $N_{5.\text{Activity-based learning}}$=1; $N_{4.\text{Simulation-based learning}}$=8; $N_{3.\text{Observation-based learning}}$=1; $N_{2.\text{Exchange-based learning}}$=9; $N_{1.\text{Theory-based learning}}$=0
